# Therapeutic Potential of Epigallocatechin Gallate Nanodelivery Systems

**DOI:** 10.1155/2017/5813793

**Published:** 2017-07-16

**Authors:** Andreia Granja, Iúri Frias, Ana Rute Neves, Marina Pinheiro, Salette Reis

**Affiliations:** UCIBIO, REQUIMTE, Department of Chemical Sciences, Faculty of Pharmacy, University of Porto, Porto, Portugal

## Abstract

Nowadays, the society is facing a large health problem with the rising of new diseases, including cancer, heart diseases, diabetes, neurodegenerative diseases, and obesity. Thus, it is important to invest in substances that enhance the health of the population. In this context, epigallocatechin gallate (EGCG) is a flavonoid found in many plants, especially in tea. Several studies support the notion that EGCG has several benefits in fighting cancer, heart diseases, diabetes, and obesity, among others. Nevertheless, the poor intestinal absorbance and instability of EGCG constitute the main drawback to use this molecule in prevention and therapy. The encapsulation of EGCG in nanocarriers leads to its enhanced stability and higher therapeutic effects. A comprehensive review of studies currently available on the encapsulation of EGCG by means of nanocarriers will be addressed.

## 1. Introduction

Green tea is an infusion of the tea plant, which has been consumed for centuries in East Asia, associated with health benefits [1, 2]. This beverage is highly concentrated in antioxidants, namely, polyphenols, such as catechins [3, 4]. Recent studies found that these polyphenols have numerous benefits in the prevention and treatment of cancer, vascular and degenerative diseases, diabetes, obesity, and other health concerns [5–7].

### 1.1. Chemical Structure of Epigallocatechin Gallate (EGCG)

**Figure 1:**
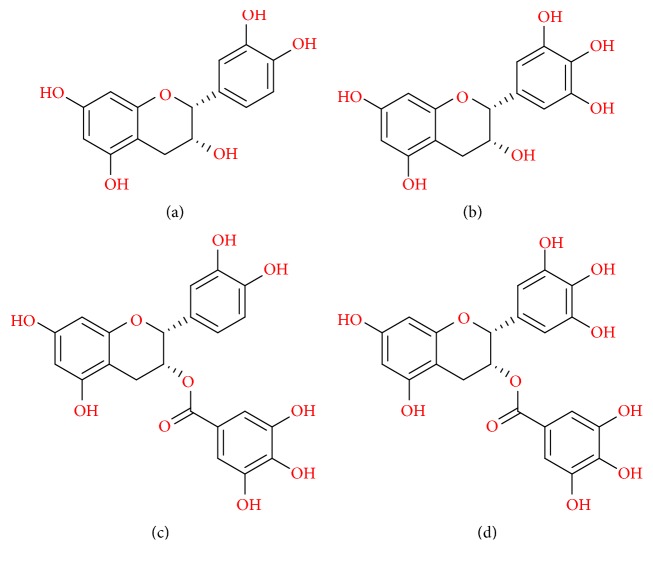
Chemical structure of the four major catechins present in tea. (a) (−)-Epicatechin (EC), (b) (−)-epigallocatechin (EGC), (c) (−)-epicatechin gallate (ECG), and (d) (−)-epigallocatechin gallate (EGCG).

Green tea has a great amount of polyphenols divided into three major groups: flavanols, flavones, and flavonols [8]. These compounds have similar chemical structure, with differences in the heterocyclic C-ring. Flavonols and flavones have a similar C-ring structure with a double bond at the 2-3 positions. The difference between the two polyphenols is the lack of a hydroxyl group at the 3-position in flavones. Flavanols differ from flavonols due to the lack of an oxygen group at the 4 position of the C-ring and the double bond at the 2-3 position [9]. Flavanols comprise the majority of the green tea content, with catechins being about one-third of the dry leaf weight, and are mainly distributed by four molecules: (−)-epicatechin (EC), (−)-epigallocatechin (EGC), (−)-epicatechin gallate (ECG), and (−)-epigallocatechin gallate (EGCG) [3]. This last one, EGCG, is the most abundant and therapeutically active catechin present in green tea, constituting 65% of the total catechin content [10]. The chemical structures of these compounds are illustrated in Figure 1.

EGCG is a complex molecule formed by a flavanol core (flavan-3-ols) structure with a gallocatechol group and a gallate ester [8]. These two gallocatechol rings confer the potent antioxidant and chelating properties to EGCG [11]. Each of the gallocatechol rings is capable of directly capturing free radicals from the environment with high efficiency [12].

Previous studies have shown that EGCG possesses a stronger antioxidant capacity compared with the other green tea catechins and it is also demonstrated that EGCG is more efficient in radical scavenging than vitamins E and C [13, 14].

In the human body, catechins are capable of reducing the amount of free radicals by chelating metal ions, especially the iron ion [15]. These metal ions are known to produce free radicals by Fenton's reaction [16]. They accomplish this sequestration by binding the ion to the catechol or the galloyl groups found in the structure of the catechins [17]. The number of these groups that are present in the catechin strongly influences the ion binding capacity [18]. The catechins with only one group, EC and EGC, are only capable of binding one ion [18]. On the other hand, the catechol group and galloyl group in ECG and the pyrogallol group and galloyl group are spatially distant from one another, which allows them to independently chelate two ions per molecule [18].

### 1.2. Therapeutic Potential of EGCG

For many years, the consumption of green tea has been associated with numerous health benefits [2, 19]. These properties can be directly linked with the polyphenol content of tea, more specifically with EGCG. For this reason, the study of EGCG is of utmost importance because this compound seems to prevent and also be useful in the treatment of numerous diseases like cancer and cardiovascular and neurodegenerative diseases [1, 20–22]. EGCG is a powerful antioxidant, anti-inflammatory, antibacterial, and antiviral agent and is capable of modulating some pathways, changing the metabolism of lipids [20, 23–26].

#### 1.2.1. Cancer Chemoprevention

Cancer is the end of several steps of cellular growth lesions, namely, hyperplasia, metaplasia, dysplasia, and neoplasia [59]. Each of the presented conditions is a progression in the cancer formation, culminating in the malignant neoplasia known as cancer [59, 60]. Nowadays, most modern therapies currently available for treating cancer are very expensive and toxic and have low effectiveness in treating the disease [60]. Therefore, it is urgent to investigate natural compounds like EGCG derived from green tea for the prevention and treatment of cancer and other diseases [59]. According to previous studies, EGCG is a promising molecule in the prevention and treatment of cancer [61–63]. Some anticancer properties of EGCG are attributed to its free radical scavenging properties, avoiding the damage of the cell structures induced by the free radicals [64]. Besides being antioxidant, EGCG has the ability to bind and modulate the activity of several signaling molecules related to mitosis, survival, and cellular death, moderating the cellular responses present in cancer [65]. Previous works demonstrated that EGCG is able to inhibit all of the processes involved in carcinogenesis: initiation, promotion, and progression [66, 67]. EGCG has the ability to bind to some proteins associated in molecular pathways that are misregulated in cancerous cells. Indeed, EGCG induces the suppression of two important transcription factors, tumor suppressor p53 and nuclear factor kappa-light-chain enhancer of activated B-cells (NF-kB), leading to regression of the tumors [66, 68]. To assist the growth of the tumor, new capillaries are needed to satisfy the oxygen and nutrient requirements of the cells [69–71]. The growth process of new blood vessels is called angiogenesis [69–71]. To promote formation of new capillaries, the tumor secretes signaling molecules to the surrounding tissues, especially vascular endothelial growth factor (VEGF). VEGF is directly influenced by the activity of hypoxia-inducible factor 1*α* (HIF-1*α*) and NF-kB factors, which are modulated by the presence of EGCG [69–71]. For these reasons, EGCG is able to diminish tumor angiogenesis and stall growth [72]. In addition, there is strong evidence that EGCG is capable of diminishing migration and metastasis formation of tumors [73, 74]. Previous studies report that EGCG promotes a reduction in the migration and metastasis formation of tumor cells with tumor size reduction, accomplishing a more reliable and efficient chemotherapy [75–77]. Although the single use of EGCG in chemotherapy is unlikely due to its inefficacy in completely eliminating the disease, it would be very interesting to use EGCG as an adjuvant of the cytostatic drugs [78, 79]. This synergism that has been reported in numerous in vitro, in vivo, and preclinical studies may be useful to reduce the amount of the necessary cytostatic drugs, which will reduce the side effects [78–80]. In addition, EGCG's antioxidant and anti-inflammatory properties are also useful to protect against chemotherapy side effects. Finally, the health benefits of EGCG would be advantageous in enhancing the overall condition of the patients [59, 68].

#### 1.2.2. Cardiovascular Benefits

Cardiovascular diseases have a high incidence, mainly in the developed world due to a sedentary lifestyle, poor nutrition, and ambient factors [81, 82]. A diet rich in cholesterol, fat, and sugar can lead to coronary diseases like arteriosclerosis and ischemia [81, 82]. Recent studies showed that EGCG can enhance the capillary circulation dilating the capillaries, diminishing inflammation, and interfering with the lipid absorption and digestion [82–84]. On the other hand, EGCG interferes directly with the lipid emulsion process in the lipid digestion [85]. This is achieved by direct interference in the micelle formation and by inhibiting the phospholipase A2, with this enzyme being of high importance in the lipid digestion [85]. The junction of the two processes can limit the absorption of lipids and consequently lower the amount of plasmatic lipids and cholesterol [85]. In addition, EGCG can lower cholesterol even more, stimulating its excretion through the bile. Moreover, EGCG will further improve the lipid profile by enhancing the lipid metabolism [86, 87]. This catechin can also modulate the process of platelets formation, from macrophage recruiting to macrophage uptake of cholesterol [86, 87]. This effect is internally modulated in the macrophage and externally helped by the anti-inflammatory response caused by EGCG [83]. Previous studies demonstrated that the administration of EGCG is capable of preventing the growth and also reducing the size of existing platelets. The action mechanism responsible for the anti-inflammatory property of EGCG is the direct inhibition of the phospholipase A2 [83].

#### 1.2.3. Neurodegenerative Diseases

The causes of neurodegenerative diseases like Parkinson's disease (PD) and Alzheimer's disease (AD) are still unknown, with various theories being proposed. Both diseases present clinical features, like the oxidative damage of neurons and accumulation of iron in specific brain areas [88]. Another relevant aspect is the accumulation of misfolded proteins in deposits, such as the *β*-amyloid peptide in AD that interferes with the survival of the neurons, leading to premature apoptosis [88]. Special interest has been assigned to the therapeutic role of antioxidants in such neurodegenerative diseases [89]. The neuroprotective properties of the EGCG agent are related to its antioxidant, anti-inflammatory, and iron chelating properties [89]. In addition, the blood-brain barrier (BBB) is permeable to EGCG [90]. The mechanism behind the passage of this hydrophilic compound through the BBB remains unknown [90]. In the literature, it is described that EGCG is more efficient in radical scavenging than vitamins C and E, with its iron chelating ability being useful to significantly improve the symptoms of these neurodegenerative diseases [14, 91]. According to what was mentioned above, EGCG is also a cellular modulator that interacts with various pathways. In neuronal cells, this catechin promotes cell survival responses and the inhibition of cell death signals, leading to an enhancement of neuronal health [21, 92, 93]. Modifications in cell signaling also promote the nonamyloid *α*-secretase pathway, diminishing the production of A*β*-amyloid peptides [91].

Several research studies confirm that EGCG has neuroprotective properties in humans, promoting an enhancement of the degree of cognition after oral administration. These studies also confirm that EGCG induces an overall increase in the cerebral activity and calmness [94].

#### 1.2.4. Infectious Diseases

Nowadays, the main strategy to fight viruses is immunization. Unfortunately, several viral infections lack one efficient vaccine, with the HIV infection being the most important. Nance et al. have shown strong HIV inhibition promoted by EGCG in cell cultures in a dose dependent manner [95]. Moreover, Li et al. have also proven that EGCG inhibit reverse transcriptase and act synergistically with another reverse transcriptase inhibitor, namely, azidothymidine [96]. Some studies also described that EGCG is capable of binding to CD4 cells, preventing the virus from anchoring and entering the host [95].

EGCG is also useful in the inhibition of other viruses, such as enterovirus 71, hepatitis C, adenovirus, herpes simplex virus, and influenza virus [24, 97–100]. One of the molecular targets that seem to be deregulated by the viral infection is the NF-kB and the MAP-kinases pathway [101–105]. As a consequence, EGCG may induce an essential immune response, which helps to fight the viral infection. Concerning antibacterial and antifungal activities, EGCG seems to be less effective in combating infectious diseases caused by bacteria and fungi [106]. The most relevant studies in the literature show that there may be some synergistic effects on EGCG association with antibiotics against multidrug-resistant strains, such as* Staphylococcus aureus* and* Stenotrophomonas maltophilia* [107, 108]. The antifungal activity of EGCG was also reported against human-pathogenic yeasts, such as* Candida albicans*. However, the mechanisms of action are still unclear [109, 110].

#### 1.2.5. Chronic Inflammatory Disorder

Inflammation is a body response to foreign structures to the human body and damage in the tissues [111]. However, in chronic inflammatory disorders, this inflammatory response is continuously active leading to the destruction of healthy tissues causing all the above-mentioned symptoms. These conditions can be incurable and cause major discomfort to the patients [88]. Rheumatoid arthritis is one chronic inflammatory disorder characterized by cellular infiltration and proliferation of the synovium, leading to the progressive destruction of the joints through the interaction between infiltrating cells and mediators [112, 113]. These injuries lead to chronic pain affecting the life quality of the patients [112]. In this disease, the cartilage cells (i.e., chondrocytes) enter in apoptosis in response to oxidative stress and some inflammatory cytokines, interleukin (IL-1*β*) and tumor necrosis factor-*α* (TNF-*α*) [112]. The same cytokines also lead to the increase of bone reabsorption and the differentiation of osteoclasts [112]. In addition, IL-1*β* is capable of increasing the amounts of reactive oxygen species via overexpression of inducible nitric oxide synthase and increases the inflammation by overexpression of cyclooxygenase (COX-2) [114]. The presence of IL-1*β* can also activate the expression of matrix metalloproteinases (MMPs) responsible for matrix degradation [114]. TNF-*α* also plays an important role in bone turnover. In arthritis, there is overexpression of TNF-*α*, which is responsible for the differentiation and activity of osteoclasts. The long-term activation of these cells leads to bone erosion and fragility [115]. The current treatment for arthritis is by the administration of methotrexate combined with analgesics and nonsteroidal anti-inflammatory drugs which can be proficient in most cases, but ineffective in some patients [113]. Moreover, recent studies have shown that this treatment tends to lose efficacy over time [113]. For this reason, new therapies are needed and EGCG may be a promising compound. In fact, EGCG has a high antioxidant activity and also capacity to decrease the inflammation response in the body [116–118]. In cartilage cell cultures, EGCG showed marked inhibition of IL-1*β* inducible nitric oxide synthase COX-2 expression and activity [119]. The expressions of both enzymes are mediated by NF-kB, which is also suppressed in the presence of EGCG [119].

#### 1.2.6. Obesity

Obesity is a medical condition characterized by excess accumulation of fat in the body in an extension that may have negative effects on the overall health condition and may lead to the development of diseases, such as diabetes and arteriosclerosis [20, 26, 120]. The main treatment of obesity is lifestyle reeducation, including diet modification [121]. However, in some cases, drugs and supplements are needed to help in the process of losing weight [121]. As previously stated, EGCG interferes directly with the lipid digestion by the inhibition of the phospholipase A2 and interfering with the lipid/cholesterol emulsion in the gut [85, 122]. The lipid blocking capacity of EGCG can be highly relevant in the loss of weight and weight managing protocols. In addition, EGCG is capable of enhancing the lipid metabolism, leading to more caloric burn and consequent fat loss. EGCG can also interfere with the digestion of starch by inhibition of *α*-amylase [123]. Besides that, the ingestion of EGCG during a weight loss program is very useful because its administration is strongly linked with circulation improvement, free radical scavenging, and mood enhancement [94].

#### 1.2.7. Diabetes

EGCG has been associated with the prevention and reversion of diabetes mellitus through a number of effects, such as improvement of insulin secretion, regulation of glucose uptake, inhibition of insulin resistance, and enhancement of glucose tolerance and its role in oxidative stress and inflammation [1, 124]. However, these beneficial effects in diabetes are not regulated by a single mechanism, but still EGCG appears to act through multiple signaling pathways. Green tea intake has been reported to exert beneficial intestinal effects increasing the blood EGCG levels which in turn seem to inhibit cellular glucose uptake, improving its tolerance in vivo [124, 125]. Several studies demonstrated that EGCG significantly enhances glucose tolerance in rodents with type 2 diabetes mellitus [126–128]. Another study suggested that EGCG increases glucose-stimulated insulin secretion in db/db mice, through its potent antioxidant effect [129]. At the same time, EGCG induces tyrosine phosphorylation of insulin receptors, thereby mimicking insulin in H4IIE rat hepatoma cells [130]. In H4IIE cells, EGCG downregulates genes involved in gluconeogenesis and the synthesis of fatty acids, triacylglycerol, and cholesterol and glucokinase mRNA expression was upregulated in the liver of db/db mice in a dose dependent manner [126]. Moreover, Cai et al. showed that EGCG improves the insulin secretory function in rat pancreatic *β*-cell lines under conditions of glucotoxicity through mediation of Akt signaling pathway [131, 132]. EGCG also revealed effects on fatty acid-induced insulin resistance in skeletal muscle, through the activation of protein kinase C (PKC) or c-Jun N-terminal kinase (JNK) signaling pathways [133]. Furthermore, EGCG can also enhance AMPK/ACC cascade that blocks insulin receptor substrate-1 (IRS-1) serine phosphorylation, which is essential for the glucose uptake in response to insulin stimulus [129, 133, 134].

## 2. Nanocarriers Used to Deliver EGCG

Nanoparticles are structures that have at least one of the dimensions in the nanoscale range, and because of their high surface area to volume ratio, they present chemical, physical, and biological properties distinct from conventional materials [135]. Their versatility makes them excellent drug carriers because they can be modified in various parameters, including size and chemical composition, modifying the outer layer with different ligands to assign specificity to certain cells and/or structures among others [135]. In addition, nanocarriers can modify the pharmacokinetics and the stability of some drugs [135]. This is particularly true in the case of EGCG, where nanotechnology can be used to considerably increase the bioavailability of this catechin [136]. There are several nanosystems used in EGCG delivery, including lipid nanoparticles, liposomes, polymeric nanoparticles, gold nanoparticles, inorganic nanocarriers, and protein/peptide-based nanocarriers. From the above-mentioned nanocarriers, lipid and polymeric nanocarriers are the most extensively used for the delivery of EGCG.

### 2.1. Lipid Nanoparticles

Lipid nanoparticles were introduced in the early 1990s, presently being one of the most used nanosystems for drug delivery [137]. These nanocarriers are composed of a lipid matrix [137] possessing many advantages including physical stability, controlled release properties, high drug load, and excellent tolerability [138]. Their synthesis requires at least three components, namely, the hydrophobic lipid phase, one emulsifier, and the hydrophilic aqueous phase [139]. There are two main types of lipid nanocarriers: solid lipid nanoparticles (SLNs) and nanostructured lipid carriers (NLCs). These two lipid nanocarriers differ in the type of the lipid used; SLNs are composed of solid lipids while NLC comprise a mixture of solid and liquid lipids [139]. Due to their distinctive lipid crystalline structure, these nanocarriers have different encapsulation efficiency and release behavior. In comparison to NLC, the lipid matrix of SLN has a more ordered fully crystallized structure and for that reason tends to have a lower drug loading [139]. It also tends to expulse the drug content during long storage due to crystalline rearrangement [139]. On the other hand, NLCs have higher drug loading and storage stability in comparison to SLNs because of the higher number of imperfections in the lipid core promoted by the presence of the liquid lipid, which contributed to the creation of a more disorganized lipid matrix with more cavities available to allocate the drug [139]. Lipid nanoparticles have been used to enhance the stability of EGCG in physiological environments, to promote sustained release of a compound, and to improve its oral bioavailability. A large variety of diseases have been addressed using EGCG-loaded lipid nanocarriers including atherosclerosis and neurodegenerative, skin, and ocular diseases. The main results regarding the use of lipid nanoparticles as EGCG carriers including nanocarrier type, size, loading capacity, loading efficiency, and in vitro and in vivo evaluation are summarized in Table 1.

Zhang et al. synthetized chitosan-coated NLCs made of natural lipids (glyceryl tridecanoate and glyceryl tripalmitate) and two surfactants (soy lecithin and Kolliphor® HS15) aiming to prevent and reverse atherosclerotic lesion development through decreasing macrophage cholesterol content. The NLC formulation increases the EGCG stability and increases the cell uptake, leading to a 9-fold decrease in the accumulation of cholesterol in macrophage cultures in combination with diminishing secretion of inflammatory factors [27]. More recently, the same authors synthesized a triglyceride-free formulation for EGCG encapsulation, replacing triglyceride by alpha-tocopherol acetate, and similar results were obtained. Nanovehicles enhanced EGCG stability and accumulation inside the macrophages cytosol and promoted a reduction in the production of inflammatory factor MCP-1 [28]. Both results highlight the potential role of EGCG-loaded nanocarriers in the prevention of atherosclerosis.

Smith and coworkers demonstrated that nanolipidic particles significantly enhanced EGCG's oral bioavailability in vivo compared to free EGCG and increased its absorption into the systemic circulation. EGCG-loaded nanocarriers were also able to promote *α*-secretase activity in neuronal cells. These results suggest that EGCG may have an important role in the prevention and treatment of neurodegenerative diseases [29].

Recently, Chen et al. [30] developed lipid nanoparticles for encapsulation of various active compounds, including EGCG, for skin care applications. Lipid nanoparticles presented high uniformity and were able to successfully encapsulate hydrophobic and hydrophilic compounds both individually and in combination. Photostability studies, however, revealed that nanocarriers were not able to protect EGCG against photodegradation under UVA radiation after 168 hours of exposure.

Radhakrishnan et al. [31] produced SLN as delivery vehicle of EGCG to enhance its stability and anticancer effects. SLNs were successful in increasing EGCG's stability in serum and physiological conditions and provided sustained release of the compound at pH 5. In vitro studies using breast cancer cells MDA-MB-231 and prostate cancer cells DU-145 revealed a sharp decrease in cell viability and higher levels of apoptosis comparing to free EGCG.

EGCG has also been explored for the treatment of ocular diseases related to oxidative stress and ROS production such as diabetic macular edema or glaucoma. Recently, Fangueiro et al. [32] synthesized EGCG-loaded cationic lipid nanoparticles for ocular delivery. Nanocarriers induced prolonged release of EGCG in physiological medium and were able to induce EGCG permeation across rabbit cornea and sclera. Additionally, EGCG-loaded formulations were found to be tolerable and nonirritant. These results highlight the potential of the produced drug delivery vehicles for ocular delivery of EGCG.

### 2.2. Liposomes

Liposomes are phospholipid vesicles composed of at least one lipid bilayer and an aqueous milieu [140, 141]. Liposomes are versatile nanocarriers because of their amphiphilic structure with both hydrophilic and hydrophobic environments, which enables the entrapment of hydrophobic, hydrophilic, and amphiphilic drugs [140, 141]. The size of liposomes can be finely adjusted and their surface can be chemically modified to target specific tissues or evade the immune system [140, 141]. External factors, such as temperature, pH, light, and enzymes presence, can trigger abrupt release of liposomes' content by interfering with the membrane stability, causing lipid layer disruption and immediate drug release [140, 141]. Despite the promising results of liposomes as nanocarriers, few studies were found using them as EGCG carriers for therapeutic applications as shown in Table 2.

Gharib and coworkers synthesized EGCG-loaded nanoliposomes made of egg lecithin and cholesterol and evaluated their activity against methicillin-resistant* Staphylococcus aureus *(MRSA). The results showed that EGCG encapsulation into cationic nanoliposomes enhanced its effect against MRSA burn wound infections compared to free EGCG both in vitro and in vivo. These results showed that EGCG is an efficient antibacterial agent [33].

Luo et al. [34] synthetized liposomes made of phosphatidylcholine (PhC)/cholesterol aiming to prevent carcinogenesis. The produced liposome demonstrated high stability in gastric conditions with a small cargo loss of around 20%, which increases slightly in the intestinal fluid to 40%. In addition, the formulation enhanced the inhibitory effect of EGCG on tumor cell viability at a higher concentration.

Song et al. synthesized liposomes with nonionic surfactants and cholesterol to improve the oral absorption of EGCG. The authors also investigated the permeability of Caco-2 cell monolayers to the EGCG entrapped liposomes in comparison with free EGCG. The liposomes were capable of significantly enhancing the apparent permeability of EGCG. Compared with the free drugs, the formulation exhibited better stability and lower toxicity [35]. These promising results stimulate the investigation of a liposome-based nanoformulation to enhance the bioavailability of EGCG by increasing its intestinal permeability.

More recently, Ramadass et al. [36] synthesized a liposome for coloading of EGCG and paclitaxel for breast cancer therapy. The produced nanoformulation could effectively potentiate the synergistic effect between the two compounds and promote breast cancer cell apoptosis in MDA-MB-231 cells.

### 2.3. Polymeric Nanocarriers

Polymeric nanocarriers are made of polymers, which can be natural or synthetic [142]. In polymeric nanocarriers, the drug is entrapped in the polymer matrix, being protected from the outside environment. To enhance the pharmacokinetic profile of the drug, or in some cases to target some specific tissue, the outer layer of the polymer can be functionalized using molecular markers [142]. There are several papers reporting the use of polymeric nanocarriers to deliver EGCG in vitro and in vivo with promising results [142]. These studies are summarized in Table 3.

Chitosan has been the most popular choice to encapsulate EGCG regarding its biocompatibility, biodegradability, and low toxicity [143]. Moreover, chitosan is mucoadhesive and can enhance the permeability of the intestine by opening the tight junctions [143]. Dube et al. used chitosan nanocarriers to enhance the intestinal absorption of EGCG in vitro [37]. Moreover, the same authors proved that chitosan nanocarriers enhance the EGCG uptake in vivo [37, 38]. In a similar way, Hu et al. synthetized chitosan nanovehicles cross-linked with casein phosphopeptides. The developed nanocarriers enhance the stability of EGCG in the gastrointestinal system [39]. In addition, the permeability of EGCG was increased in Caco-2 cells monolayers [40]. Hong et al. synthesized EGCG-loaded chitosan and aspartic acid self-assembled nanocarriers. These nanoformulations significantly improved EGCG antiatherosclerotic effects in vivo after oral administration to rabbits [41]. Khan and coworkers used EGCG-loaded chitosan nanocarriers in athymic mice implanted with prostate cancer cells and found a reduction in the growth of the tumor compared to the two controls, one that received the nanocarriers and the other that received the free EGCG. The results also indicate a dose dependent relation between the amount of encapsulated EGCG and the size of the tumor [42]. Recently, Zeng and coworkers [43] synthesized chitosan tripolyphosphate (CS/TPP) nanocarriers modified with folic acid (FA) and poly(ethylene glycol) (PEG) for EGCG encapsulation. Nanocarriers promoted a decrease in MCF-7 breast cancer cell proliferation compared to the control group. This effect was further increased by modification with FA and PEG. Cell uptake was also induced, particularly for nanocarriers modified with FA, demonstrating the efficacy of the functionalization. Finally, encapsulated EGCG could induce higher levels of modulation of the PI3K-Akt pathway and exert its antiproliferative effects.

Polylactic acid (PLA) and poly(lactide-co-glycolide) acid (PLGA) are two of the most extensively studied polymers to create nanocarriers due to their versatility and high biocompatibility [142, 144]. The outer layer of these nanocarriers can be easily modified with functional groups to specifically target certain cells and/or structures. In addition, the degradation over time of these nanocarriers can also be manipulated and they can be synthetized to have high encapsulation rates and controlled release properties [142, 144]. Sanna et al. synthetized PLGA-based nanocarriers coated with PEG and functionalized with a prostate-specific membrane antigen (PSMA) inhibitor for the chemoprevention of prostate cancer. The nanocarriers were tested in prostate cancer cell lines with promising results and induced higher bioavailability of EGCG [44]. Siddiqui et al. also had encouraging results but instead of PLGA-PEG they used PLA-PEG nanocarriers for nanochemoprevention [45]. Sanna et al. [46] recently published an updated paper where PLGA-PEG nanocarriers functionalized with two different small peptides specific for PSMA were synthesized. In vitro results demonstrated, once again, an accentuated antiproliferative effect on different prostate cancer cell types compared to that of free EGCG. These results were then corroborated by performing in vivo studies in an athymic nude mice xenograft model. Significant inhibition of tumor growth was obtained compared to that of free EGCG. In addition, targeted nanocarriers were more effective in inhibiting tumor growth, demonstrating the efficacy of the functionalization.

Srivastava et al. used EGCG-loaded PLGA nanocarriers to prevent DNA damage and to be used in chemoprevention. In this study, mice were treated topically with EGCG and PLGA EGCG nanocarriers prior to the damage of DNA. Pretreatment with free EGCG resulted in the protection against DNA damage in the order of 28%. Pretreatment with the EGCG-loaded nanocarriers resulted in 63% protection from DNA damage. Moreover, EGCG-loaded nanocarriers showed significant induction of DNA repair genes and inhibition of inflammatory genes [47]. Despite their beneficial properties, PLA and PLGA polymers have the drawback of being unstable in acidic environments which does not make them suitable for oral administration [44, 45]. Li and Gu used a different type of polymer, namely, ovalbumin-dextran, to produce nanocarriers aiming to improve stability in the gastrointestinal tract and to enhance the absorption of loaded EGCG. The results revealed that the ovalbumin-dextran nanocarriers were stable in simulated gastric and intestinal fluids and the absorption profile of EGCG was improved since the formulation enhanced the apparent permeability coefficient (*P*_app_) of EGCG on Caco-2 monolayers compared with free EGCG [48].

### 2.4. Gold Nanocarriers

Gold nanoparticles are made from a gold core covered with a film of functional molecules, which are capable of transporting complex molecules, including drugs and targeting molecules [49]. Various reports, presented in Table 4, have described the use of gold nanoparticles as delivery vehicles of EGCG. It is possible to synthetize gold nanocarriers covered with EGCG due to the capacity of this catechin to combine with metals, forming complexes by the gallate ring [49]. Although gold EGCG complexes present high stability in acidic pH conditions, they are unstable in alkaline conditions. To circumvent this disadvantage, Shukla et al. and Chen et al. proposed the intratumoral administration [50, 51]. Shukla and coworkers synthetized radioactive gold nanocarriers coated with EGCG to be used in the treatment of prostate and other solid tumors. In this case, EGCG served as a chemoadjuvant and targeting molecule simultaneously, due to its anticancer properties and high affinity and specificity to the Laminin 67R receptor which is overexpressed in several cancers including prostate cancer cells. Therefore, by coating gold nanocarriers with EGCG, it is possible to target prostate cancer cells and promote internalization by receptor-mediated endocytosis. The results obtained were very promising and a high efficacy of the formulation was achieved in reducing the tumor size in the mice [50]. On the other hand, Chen et al. developed nonradioactive gold nanocarriers coated with EGCG to inhibit the appearance and the development of tumors [51]. The authors found a promising cytotoxic effect both in vitro and in vivo using a murine melanoma cancer model. The nanocarriers showed improved anticancer efficacy in murine melanoma cells, promoting cytotoxic effects 4.91 times higher than those treated with free EGCG. Despite these promising results, the two gold nanoformulations mentioned above were administered by intratumoral injection, which is an invasive route of administration. Hsieh et al. synthetized gold nanocarriers to increase the gastrointestinal stability of EGCG. The obtained nanosystems were revealed to be stable in the gastrointestinal environment with sustained release of EGCG over 2 hours. The authors also demonstrated that the formulation showed preferential toxicity to cancerous cells and an in vivo efficiency in reducing the growth of implanted tumors in mice. The sustained release and the high cytotoxicity to cancerous cells make these nanocarriers promising future nanoformulations to use in cancer therapy [52]. Recently, EGCG conjugated gold nanoparticles have also been employed for the treatment of cardiovascular diseases. Khoobchandani and coworkers [53] developed gold nanocarriers as an alternative to drug coated stents. Nanocarriers were stable in several biological media and were internalized in both smooth muscle and endothelial cells via Laminin 67R receptor-mediated endocytosis. In addition, EGCG-Au nanocarriers inhibited the migration of smooth muscle cells, while maintaining endothelial cells' viability and proliferation capacity. These results revealed the efficiency of EGCG-coated gold nanocarriers for the treatment of neointimal hyperplasia and restenosis, highlighting the potential of these nanovehicles for the treatment of cardiovascular diseases.

### 2.5. Other Nanocarriers

Numerous other different materials can be employed for the development of nanocarriers for drug delivery. Some of these nanocarriers used for EGCG entrapment are summarized in Table 5.

Zhang et al. synthesized EGCG-stabilized selenium nanocarriers coated with Tet-1 peptide as a potential therapy for neurodegenerative diseases. Selenium nanocarriers revealed high affinity to amyloid beta (A*β*) and elevated capacity to inhibit A*β* fibrillation and to promote A*β* fibril disaggregation. In addition, these nanocarriers were able to protect PC12 cells against A*β*-mediated toxicity. The results demonstrated that incorporation of EGCG into nanocarriers increased its therapeutic efficacy in neurodegenerative diseases [54]. EGCG's strong antioxidant effects can also be exploited for skin care, providing antiaging benefits and protection against UV radiation. In this context, Avadhani et al. [55] synthesized and optimized transfersomes for coentrapment of EGCG and hyaluronic acid. The produced formulation demonstrated free radical scavenging effects, ability to suppress lipid peroxidation, ROS generation, and MMPs expression in human keratinocyte cell line (HaCaT). In addition, transfersomes were able to increase skin permeation and deposition of EGCG compared to free EGCG. Similar results were obtained by Shetty et al. [56]. In this work, peptide dendrimers were produced to enhance the transdermal delivery of silibinin and EGCG. Peptide dendrimers could increase skin penetration and deposition of both compounds, thus highlighting their suitability as delivery vehicles for skin care applications including antiaging and UV radiation protection.

EGCG antiangiogenic properties have recently been explored for the inhibition of corneal neovascularization as a therapy for several ocular diseases such as blindness. Chang et al. [57] developed gelatin/EGCG nanocarriers coated with arginine-glycine-aspartic acid (RGD) peptide to target the *α*_v_*β*_3_ integrin expressed on human umbilical vein endothelial cells (HUVECs) in order to inhibit their activity and thus suppress angiogenesis. The produced nanocarriers could effectively target the *α*_v_*β*_3_ integrin and inhibit HUVECs proliferation and migration in comparison to free EGCG. These antiangiogenic effects were further confirmed in an in vivo study using a corneal neovascularization mouse model, where a reduction of vessel growth in the cornea was observed.

Fan et al. [58] produced *β*-lactoglobulin-chlorogenic acid nanocarriers for EGCG encapsulation. These nanocarriers could effectively increase EGCG's chemical stability in physiological environments and induce controlled release of EGCG in simulated gastric and intestinal environments, being able to protect EGCG from degradation.

## 3. Conclusion

There is evidence that tea catechins and, specially, EGCG contribute to the prevention and treatment of several health problems, including cancer, Parkinson's disease, Alzheimer's disease, obesity, and cardiovascular diseases. EGCG is nontoxic with no documented side effects, making this natural compound a popular choice for research and for the prevention and treatment of several diseases. However, this polyphenol compound possesses low bioavailability and consequently a large dose is required to reach therapeutic concentrations. Several studies have applied the use of nanocarriers to improve the bioavailability and stability of EGCG. Different types of nanocarriers have been used, including lipid nanoparticles, liposomes, polymeric nanoparticles, gold nanoparticles, and other types of nanocarriers composed of inorganic materials, proteins, and peptides. The majority of the studies have focused on lipid and polymeric nanocarriers, possibly due to their beneficial properties such as biocompatibility. On the other hand, the safety of gold and inorganic nanocarriers remains uncertain. The results are very promising and the nanocarrier formulations increased the bioavailability and stability of EGCG. Moreover, some studies showed that the nanoformulations could increase the permeability in the intestinal barrier with impressive results when the particles were decorated with ligands, such as chitosan, which is mucoadhesive and opens the tight junctions of the intestine. Additionally, the incorporation of target ligands on the surface of the nanocarriers could increase the delivery of EGCG to targeted abnormal cells, including cancer cells. Overall, nanocarriers could efficiently potentiate the therapeutic activities of EGCG in the treatment of numerous disorders including cancer and neurodegenerative, skin, and ocular diseases.

## 4. Future Directions

The cost of applying nanotechnology is a major limitation and, for that reason, it is important to lower the cost/benefit for the application of nanocarriers as drug delivery systems for prevention and therapeutic purposes [145]. In this context, lipid nanocarriers seem to be very promising since they are inexpensive and easy to scale up. These types of nanocarriers showed impressive results in terms of loading efficiency, which is essential to reduce the costs and the toxicity associated with the excipients. The high encapsulation efficiency obtained is due to the ability of this hydrophilic compound to complex with the lipids. These recent advances in EGCG nanodelivery systems reinforced the importance of nanotechnology to improve the chemoprevention and therapeutic effects of EGCG, holding a great promise for future clinical applications.

## Supplementary Material

Figure S1. Different types of nanoparticles used as delivery vehicles of EGCG.

## Figures and Tables

**Table 1:** Lipid nanoparticles used as EGCG carriers. *Note*. N/A denotes “not available” data.

Particle	Loading capacity (%)	Loading efficiency (%)	Size (nm)	Administration route	In vitro/in vivo results	Ref.
NLCs (glyceryl tridecanoate, glyceryl tripalmitate, soy lecithin and Kolliphor HS15, and chitosan)	3	99	50	Oral	High stability in both acidic and neutral environments. In vitro studies performed in THP-1-derived macrophages showed a decrease in inflammation and accumulation of cholesterol.	[27]

NLCs (phosphatidylcholine, Kolliphor HS15, and alpha-tocopherol acetate)	10	96	108	N/A	Increased EGCG stability. Enhanced accumulation inside macrophages and decrease in the production of MCP-1.	[28]

Phosphatidylcholine, phosphatidylethanolamine, phosphatidic acid, and phosphatidylinositol	N/A	N/A	30–80	Oral	Enhanced EGCG oral bioavailability in vivo and induction of *α*-secretase activity in neuronal cells in vitro.	[29]

NLCs (cetyl palmitate/Phospholipon 80, sesame oil, and Tween-80)	2.7–3.6	99	126–167	Topical	Photodegradation of EGCG under UVA radiation.	[30]

SLN (glycerol monostearate, stearic acid, soya lecithin, and Pluronic F68)	N/A	67	157	N/A	Enhanced stability in physiological fluids. Increased induction of cell death in breast cancer cells MDA-MB-231 and prostate cancer cells DU-145.	[31]

Cationic lipid nanocarriers (Softisan® 100, Poloxamer 188, glycerol, Lipoid® S75, and CTAB/DDAB)	N/A	N/A	~150	Ocular	Prolonged release of EGCG in biological medium. Permeation of rabbit cornea and sclera.	[32]

**Table 2:** Liposomes used as EGCG carriers. *Note*. N/A denotes “not available” data.

Particle	Loading capacity (%)	Loading efficiency (%)	Size (nm)	Administration route	In vitro/in vivo results	Ref.
Egg lecithin and cholesterol	N/A	45–78	89–93	Topical	Enhanced anti-MRSA activity in vitro and in vivo	[33]

PhC and cholesterol	N/A	85.8 ± 1.65	180	Oral	Favorable release profile in the gastrointestinal fluids.	[34]

Sorbitan monostearate and cholesterol	N/A	40	100	Oral	The nanoformulation presents a high stability in neutral pH and enhances the cellular permeability in caco-2 cell monolayer.	[35]

Cholesterol, phosphatidylcholine, and Tween-80	60.21 ± 1.59	N/A	126.7 ± 4.3	N/A	Enhancing synergistic effects between EGCG and paclitaxel in inducing apoptosis in MDA-MB-231 breast cancer cells.	[36]

**Table 3:** Polymeric nanoparticles used as EGCG carriers. *Note*. N/A denotes “not available” data.

Particle	Loading capacity (%)	Loading efficiency (%)	Size (nm)	Administration route	In vitro/in vivo results	Ref.
Chitosan	0.4	N/A	440 ± 37	Oral	Enhancement of the gastrointestinal permeation of EGCG in mice.	[37, 38]

Chitosan, casein, and peptides	N/A	N/A	150	Oral	Bioavailability of EGCG increment in Caco-2 monolayers.	[39]

Chitosan casein phosphopeptides	N/A	N/A	150 ± 4.3	Oral	Enhancement of the intestinal permeation of EGCG using Caco-2 monolayers.	[40]

Chitosan and aspartic acid	N/A	25	102	Oral	Increased antiatherosclerotic activity in rabbits.	[41]

Chitosan	N/A	~10	~150	Oral	Reduction of human prostate tumors in mice.	[42]

Chitosan tripolyphosphate (CS/TPP)	N/A	40–90	143–450	N/A	Inhibition of MCF-7 breast cancer cells proliferation. Higher levels of modulation of PI3K-Akt pathway.	[43]

PLGA and PEG	~0,4	~9,5	80 ± 15.0	Intravenous	Inhibition of the growth of cultured cancerous cells.	[44]

PLA PEG	N/A	N/A	N/A	Intravenous	Reduction in the size of the implanted tumor in mice.	[45]

PLGA and PEG	1.94–2.21	49–55	130–250	Intravenous	Accentuated antiproliferative effect on 3 different prostate cancer cell types. Significant inhibition of prostate tumor growth in vivo.	[46]

PLGA	5.76	26	127	Topical	Inhibition of DNA damage.	[47]

Ovalbumin-dextran	20.9	30.0	339	Oral	Enhancement of the intestinal stability and improvement of apparent permeability in Caco-2 models.	[48]

**Table 4:** Gold nanoparticles used as EGCG carriers. *Note*. N/A denotes “not available” data.

Particle	Loading capacity (%)	Loading efficiency (%)	Size (nm)	Administration route	In vitro/in vivo results	Ref.
Gold	N/A	N/A	20–1200	Intratumoral	Noticeable reduction in bladder tumor in mice.	[49]

Radioactive gold	N/A	N/A	N/A	Intratumoral	Noticeable reduction of tumor size achieved after 28 days with a single administration of the formulation and with minor radioactive leakage to other organs.	[50]

Gold	2	27	68	Intratumoral	High cytotoxicity in melanoma cell culture and in mice.	[51]

Gold	N/A	27	50	Oral	Nanoformulation stable in neutral pH with sustained release of 2 hours. The in vitro and in vivo experiments revealed that the nanoformulation is highly effective in the cancer therapy.	[52]

Gold	N/A	N/A	65	N/A	Selective inhibition of smooth muscle cell migration.	[53]

**Table 5:** Other EGCG nanocarriers. *Note*. N/A denotes “not available” data.

Particle	Loading capacity (%)	Loading efficiency (%)	Size (nm)	Administration route	In vitro/in vivo results	Ref.
Selenium	N/A	N/A	29	N/A	Enhanced capacity to inhibit A*β* fibrillation and promote fibril disaggregation. Protection of PC-12 against A*β*-mediated toxicity.	[54]

Transfersomes	N/A	76.5	101.2	Topical	Increased ability to suppress lipid peroxidation, ROS generation, and MMPs expression. Enhanced ex vivo skin permeation and deposition of EGCG across rat skin.	[55]

Peptide dendrimers (glycine, proline, lysine, and arginine)	N/A	N/A	N/A	Topical	Enhanced ex vivo skin permeation and deposition of EGCG across rat skin.	[56]

Gelatin	N/A	97.13	168.87	Ocular	Inhibition of HUVECs proliferation and migration. Inhibition of vessel formation in a corneal neovascularization mouse model.	[57]

*β*-Lactoglobulin-chlorogenic acid	~7.3	~73	~110	N/A	Enhanced EGCG stability in physiological environments. Controlled release in simulated gastric and intestinal environments.	[58]

## References

[1] Chowdhury A., Sarkar J., Chakraborti T., Pramanik P. K., Chakraborti S. (2016). Protective role of epigallocatechin-3-gallate in health and disease: a perspective. *Biomedicine and Pharmacotherapy*.

[2] Afzal M., Safer A. M., Menon M. (2015). Green tea polyphenols and their potential role in health and disease. *Inflammopharmacology*.

[3] Graham H. N. (1992). Green tea composition, consumption, and polyphenol chemistry. *Preventive Medicine*.

[4] Cirillo G., Curcio M., Vittorio O. (2016). Polyphenol Conjugates and Human Health: A Perspective Review. *Critical Reviews in Food Science and Nutrition*.

[5] Singh G., Kaur H., Harikumar S. L. (2015). Pleiotropic effects of green tea: an overview. *International Journal of Pharmaceutical and Phytopharmacological Research*.

[6] Zaveri N. T. (2006). Green tea and its polyphenolic catechins: medicinal uses in cancer and noncancer applications. *Life Sciences*.

[7] Crespy V., Williamson G. (2004). A review of the health effects of green tea catechins in in vivo animal models. *J Nutr*.

[8] Botten D., Fugallo G., Fraternali F., Molteni C. (2015). Structural Properties of Green Tea Catechins. *Journal of Physical Chemistry B*.

[9] Hollman P. C. H., Arts I. C. W. (2000). Flavonols, flavones and flavanols - Nature, occurrence and dietary burden. *Journal of the Science of Food and Agriculture*.

[10] Islam M. A. (2012). Cardiovascular effects of green tea catechins: Progress and promise. *Recent Patents on Cardiovascular Drug Discovery*.

[11] Braicu C., Ladomery M. R., Chedea V. S., Irimie A., Berindan-Neagoe I. (2013). The relationship between the structure and biological actions of green tea catechins. *Food Chemistry*.

[12] Nanjo F., Goto K., Seto R., Suzuki M., Sakai M., Hara Y. (1996). Scavenging effects of tea catechins and their derivatives on 1,1- diphenyl-2-picrylhydrazyl radical. *Free Radical Biology and Medicine*.

[13] Mukai K., Mitani S., Ohara K., Nagaoka S.-I. (2005). Structure-activity relationship of the tocopherol-regeneration reaction by catechins. *Free Radical Biology and Medicine*.

[14] Rice-Evans C. A., Miller N. J., Bolwell P. G., Bramley P. M., Pridham J. B. (1995). The relative antioxidant activities of plant-derived polyphenolic flavonoids. *Free Radical Research*.

[15] Weinreb O., Amit T., Mandel S., Youdim M. B. H. (2009). Neuroprotective molecular mechanisms of (−)-epigallocatechin-3-gallate: a reflective outcome of its antioxidant, iron chelating and neuritogenic properties. *Genes and Nutrition*.

[16] Filipský T., Mladenka P., Macáková K. (2014). Effect of novel 1-phenyl-3-methyl-4-acylpyrazolones on iron chelation and Fenton reaction. *Free Radical Biology and Medicine*.

[17] Thephinlap C., Ounjaijean S., Khansuwan U., Fucharoen S., Porter J. B., Srichairatanakool S. (2007). Epigallocatechin-3-gallate and epicatechin-3-gallate from green tea decrease plasma non-transferrin bound iron and erythrocyte oxidative stress. *Medicinal Chemistry*.

[18] Ryan P., Hynes M. J. (2007). The kinetics and mechanisms of the complex formation and antioxidant behaviour of the polyphenols EGCg and ECG with iron(III). *Journal of Inorganic Biochemistry*.

[19] Da Silva Pinto M. (2013). Tea: A new perspective on health benefits. *Food Research International*.

[20] Legeay S., Rodier M., Fillon L., Faure S., Clere N. (2015). Epigallocatechin gallate: A review of its beneficial properties to prevent metabolic syndrome. *Nutrients*.

[21] Xicota L., Rodríguez-Morató J., Dierssen M., De La Torre R. (2015). Potential role of (−)-epigallocatechin-3-gallate (EGCG) in the secondary prevention of alzheimer disease. *Current Drug Targets*.

[22] Keske M. A., Ng H. L. H., Premilovac D. (2015). Vascular and metabolic actions of the green tea polyphenol epigallocatechin gallate. *Current Medicinal Chemistry*.

[23] Farzaei M. H., Rahimi R., Abdollahi M. (2015). The role of dietary polyphenols in the management of inflammatory bowel disease. *Current Pharmaceutical Biotechnology*.

[24] Hsu S. (2015). Compounds derived from epigallocatechin-3-gallate (EGCG) as a novel approach to the prevention of viral infections. *Inflammation and Allergy—Drug Targets*.

[25] Reygaert W. C. (2014). The antimicrobial possibilities of green tea. *Frontiers in Microbiology*.

[26] Yang C. S., Zhang J., Zhang L., Huang J., Wang Y. (2016). Mechanisms of body weight reduction and metabolic syndrome alleviation by tea. *Molecular Nutrition and Food Research*.

[27] Zhang J., Nie S., Wang S. (2013). Nanoencapsulation enhances epigallocatechin-3-gallate stability and its antiatherogenic bioactivities in macrophages. *Journal of Agricultural and Food Chemistry*.

[28] Zhang J., Nie S., Martinez-Zaguilan R., Sennoune S. R., Wang S. (2016). Formulation, characteristics and antiatherogenic bioactivities of CD36-targeted epigallocatechin gallate (EGCG)-loaded nanoparticles. *Journal of Nutritional Biochemistry*.

[29] Smith A., Giunta B., Bickford P. C., Fountain M., Tan J., Shytle R. D. (2010). Nanolipidic particles improve the bioavailability and *α*-secretase inducing ability of epigallocatechin-3-gallate (EGCG) for the treatment of Alzheimer's disease. *International Journal of Pharmaceutics*.

[30] Chen J., Wei N., Lopez-Garcia M. (2017). Development and evaluation of resveratrol, Vitamin E, and epigallocatechin gallate loaded lipid nanoparticles for skin care applications. *European Journal of Pharmaceutics and Biopharmaceutics*.

[31] Radhakrishnan R., Kulhari H., Pooja D. (2016). Encapsulation of biophenolic phytochemical EGCG within lipid nanoparticles enhances its stability and cytotoxicity against cancer. *Chemistry and Physics of Lipids*.

[32] Fangueiro J. F., Calpena A. C., Clares B. (2016). Biopharmaceutical evaluation of epigallocatechin gallate-loaded cationic lipid nanoparticles (EGCG-LNs): in vivo, in vitro and ex vivo studies. *International Journal of Pharmaceutics*.

[33] Gharib A., Faezizadeh Z., Godarzee M. (2013). Therapeutic efficacy of epigallocatechin gallate-loaded nanoliposomes against burn wound infection by methicillin-resistant staphylococcus aureus. *Skin Pharmacology and Physiology*.

[34] Luo X., Guan R., Chen X., Tao M., Ma J., Zhao J. (2014). Optimization on condition of epigallocatechin-3-gallate (EGCG) nanoliposomes by response surface methodology and cellular uptake studies in Caco-2 cells. *Nanoscale Research Letters*.

[35] Song Q., Li D., Zhou Y. (2014). Enhanced uptake and transport of (+)-catechin and (−)-epigallocatechin gallate in niosomal formulation by human intestinal caco-2 cells. *International Journal of Nanomedicine*.

[36] Ramadass S. K., Anantharaman N. V., Subramanian S., Sivasubramanian S., Madhan B. (2015). Paclitaxel/Epigallocatechin gallate coloaded liposome: a synergistic delivery to control the invasiveness of MDA-MB-231 breast cancer cells. *Colloids and Surfaces B: Biointerfaces*.

[37] Dube A., Nicolazzo J. A., Larson I. (2010). Chitosan nanoparticles enhance the intestinal absorption of the green tea catechins (+)-catechin and (−)-epigallocatechin gallate. *European Journal of Pharmaceutical Sciences*.

[38] Dube A., Nicolazzo J. A., Larson I. (2011). Chitosan nanoparticles enhance the plasma exposure of (−)-epigallocatechin gallate in mice through an enhancement in intestinal stability. *European Journal of Pharmaceutical Sciences*.

[39] Hu B., Ting Y., Yang X., Tang W., Zeng X., Huang Q. (2012). Nanochemoprevention by encapsulation of (−)-epigallocatechin-3-gallate with bioactive peptides/chitosan nanoparticles for enhancement of its bioavailability. *Chemical Communications*.

[40] Hu B., Ting Y., Zeng X., Huang Q. (2012). Cellular uptake and cytotoxicity of chitosan-caseinophosphopeptides nanocomplexes loaded with epigallocatechin gallate. *Carbohydrate Polymers*.

[41] Hong Z., Xu Y., Yin J.-F., Jin J., Jiang Y., Du Q. (2014). Improving the effectiveness of (−)-epigallocatechin gallate (EGCG) against rabbit atherosclerosis by EGCG-loaded nanoparticles prepared from chitosan and polyaspartic acid. *Journal of Agricultural and Food Chemistry*.

[42] Khan N., Bharali D. J., Adhami V. M. (2014). Oral administration of naturally occurring chitosan-based nanoformulated green tea polyphenol EGCG effectively inhibits prostate cancer cell growth in a xenograft model. *Carcinogenesis*.

[43] Zeng L., Yan J., Luo L., Ma M., Zhu H. (2017). Preparation and characterization of (−)-Epigallocatechin-3-gallate (EGCG)-loaded nanoparticles and their inhibitory effects on Human breast cancer MCF-7 cells. *Scientific Reports*.

[44] Sanna V., Pintus G., Roggio A. M. (2011). Targeted biocompatible nanoparticles for the delivery of (-)-epigallocatechin 3-gallate to prostate cancer cells. *Journal of Medicinal Chemistry*.

[45] Siddiqui I. A., Adhami V. M., Ahmad N., Mukhtar H. (2010). Nanochemoprevention: Sustained release of bioactive food components for cancer prevention. *Nutrition and Cancer*.

[46] Sanna V., Singh C. K., Jashari R. (2017). Targeted nanoparticles encapsulating (−)-epigallocatechin-3-gallate for prostate cancer prevention and therapy. *Scientific Reports*.

[47] Srivastava A. K., Bhatnagar P., Singh M. (2013). Synthesis of PLGA nanoparticles of tea polyphenols and their strong in vivo protective effect against chemically induced DNA damage. *International Journal of Nanomedicine*.

[48] Li Z., Gu L. (2014). Fabrication of self-assembled (−)-epigallocatechin gallate (EGCG) ovalbumin-dextran conjugate nanoparticles and their transport across monolayers of human intestinal epithelial caco-2 cells. *Journal of Agricultural and Food Chemistry*.

[49] Hsieh D.-S., Wang H., Tan S.-W. (2011). The treatment of bladder cancer in a mouse model by epigallocatechin-3-gallate-gold nanoparticles. *Biomaterials*.

[50] Shukla R., Chanda N., Zambre A. (2012). Laminin receptor specific therapeutic gold nanoparticles (198AuNP-EGCg) show efficacy in treating prostate cancer. *Proceedings of the National Academy of Sciences of the United States of America*.

[51] Chen C.-C., Hsieh D.-S., Huang K.-J. (2014). Improving anticancer efficacy of (−)-epigallocatechin-3-gallate gold nanoparticles in murine B16F10 melanoma cells. *Drug Design, Development and Therapy*.

[52] Hsieh D.-S., Lu H.-C., Chen C.-C., Wu C.-J., Yeh M.-K. (2012). The preparation and characterization of gold-conjugated polyphenol nanoparticles as a novel delivery system. *International Journal of Nanomedicine*.

[53] Khoobchandani M., Katti K., Maxwell A., Fay W. P., Katti K. V. (2016). Laminin receptor-avid nanotherapeutic EGCg-AuNPs as a potential alternative therapeutic approach to prevent restenosis. *International Journal of Molecular Sciences*.

[54] Zhang J., Zhou X., Yu Q. (2014). Epigallocatechin-3-gallate (EGCG)-stabilized selenium nanoparticles coated with Tet-1 peptide to reduce amyloid-*β* aggregation and cytotoxicity. *ACS Applied Materials and Interfaces*.

[55] Avadhani K. S., Manikkath J., Tiwari M. (2017). Skin delivery of epigallocatechin-3-gallate (EGCG) and hyaluronic acid loaded nano-transfersomes for antioxidant and anti-aging effects in UV radiation induced skin damage. *Drug Delivery*.

[56] Shetty P. K., Manikkath J., Tupally K. (2017). Skin delivery of EGCG and silibinin: potential of peptide dendrimers for enhanced skin permeation and deposition. *AAPS PharmSciTech*.

[57] Chang C., Wang M., Miyagawa T. (2017). Preparation of arginine&ndash;glycine&ndash;aspartic acid-modified biopolymeric nanoparticles containing epigalloccatechin-3-gallate for targeting vascular endothelial cells to inhibit corneal neovascularization. *International Journal of Nanomedicine*.

[58] Fan Y., Zhang Y., Yokoyama W., Yi J. (2017). *β*-Lactoglobulin-chlorogenic acid conjugate-based nanoparticles for delivery of (−)-epigallocatechin-3-gallate. *RSC Advances*.

[59] Singh B. N., Shankar S., Srivastava R. K. (2011). Green tea catechin, epigallocatechin-3-gallate (EGCG): mechanisms, perspectives and clinical applications. *Biochemical Pharmacology*.

[60] Barr R. D., Ferrari A., Ries L., Whelan J., Bleyer W. A. (2016). Cancer in adolescents and young adults: A narrative review of the current status and a view of the future. *JAMA Pediatrics*.

[61] Baker K. M., Bauer A. C. (2015). Green Tea Catechin, EGCG, Suppresses PCB 102-Induced Proliferation in Estrogen-Sensitive Breast Cancer Cells. *International Journal of Breast Cancer*.

[62] Toden S., Tran H.-M., Tovar-Camargo O. A., Okugawa Y., Goel A. (2016). Epigallocatechin-3-gallate targets cancer stem-like cells and enhances 5-fluorouracil chemosensitivity in colorectal cancer. *Oncotarget*.

[63] Zhou Y., Tang J., Du Y., Ding J., Liu J.-Y. (2016). The green tea polyphenol EGCG potentiates the antiproliferative activity of sunitinib in human cancer cells. *Tumor Biology*.

[64] Farhan M., Khan H. Y., Oves M. (2016). Cancer therapy by catechins involves redox cycling of copper ions and generation of reactive oxygenspecies. *Toxins*.

[65] Rahmani A. H., Al Shabrmi F. M., Allemailem K. S., Aly S. M., Khan M. A. (2015). Implications of green tea and its constituents in the prevention of cancer via the modulation of cell signalling pathway. *BioMed Research International*.

[66] Shankar S., Ganapathy S., Srivastava R. K. (2007). Green tea polyphenols: biology and therapeutic implications in cancer. *Frontiers in Bioscience*.

[67] Wang P., Aronson W. J., Huang M. (2010). Green tea polyphenols and metabolites in prostatectomy tissue: implications for cancer prevention. *Cancer Prevention Research (Philadelphia, Pa.)*.

[68] Yamauchi R., Sasaki K., Yoshida K. (2009). Identification of epigallocatechin-3-gallate in green tea polyphenols as a potent inducer of p53-dependent apoptosis in the human lung cancer cell line A549. *Toxicology in Vitro*.

[69] Li X., Feng Y., Liu J., Feng X., Zhou K., Tang X. (2013). Epigallocatechin-3-gallate inhibits IGF-I-stimulated lung cancer angiogenesis through downregulation of HIF-1*α* and VEGF expression. *Journal of Nutrigenetics and Nutrigenomics*.

[70] Sakamoto Y., Terashita N., Muraguchi T., Fukusato T., Kubota S. (2013). Effects of epigallocatechin-3-gallate (EGCG) on a549 lung cancer tumor growth and angiogenesis. *Bioscience, Biotechnology and Biochemistry*.

[71] Shi J., Liu F., Zhang W., Liu X., Lin B., Tang X. (2015). Epigallocatechin-3-gallate inhibits nicotine-induced migration and invasion by the suppression of angiogenesis and epithelial-mesenchymal transition in non-small cell lung cancer cells. *Oncology Reports*.

[72] Gu J.-W., Makey K. L., Tucker K. B. (2013). EGCG, a major green tea catechin suppresses breast tumor angiogenesis and growth via inhibiting the activation of HIF-1*α* and NF*κ*B, and VEGF expression. *Vascular Cell*.

[73] Chang C.-W., Hsieh Y.-H., Yang W.-E., Yang S.-F., Chen Y., Hu D.-N. (2014). Epigallocatechingallate inhibits migration of human uveal melanoma cells via downregulation of matrix metalloproteinase-2 activity and ERK1/2 pathway. *BioMed Research International*.

[74] Zapf M. A. C., Kothari A. N., Weber C. E. (2015). Green tea component epigallocatechin-3-gallate decreases expression of osteopontin via a decrease in mRNA half-life in cell lines of metastatic hepatocellular carcinoma. *Surgery (United States)*.

[75] Mukhtar H., Ahmad N. (2000). Tea polyphenols: prevention of cancer and optimizing health. *The American Journal of Clinical Nutrition*.

[76] Maruyama T., Murata S., Nakayama K. (2014). (−)-Epigallocatechin-3-gallate suppresses liver metastasis of human colorectal cancer. *Oncology Reports*.

[77] Takahashi A., Watanabe T., Mondal A. (2014). Mechanism-based inhibition of cancer metastasis with (−)-epigallocatechin gallate. *Biochemical and Biophysical Research Communications*.

[78] Lecumberri E., Dupertuis Y. M., Miralbell R., Pichard C. (2013). Green tea polyphenol epigallocatechin-3-gallate (EGCG) as adjuvant in cancer therapy. *Clinical Nutrition*.

[79] Wang X., Jiang P., Wang P., Yang C. S., Wang X., Feng Q. (2015). EGCG enhances Cisplatin sensitivity by regulating expression of the copper and cisplatin influx transporter CTR1 in ovary cancer. *PLoS ONE*.

[80] Cai Y., Zhang J., Chen N. G. (2016). Recent Advances in Anticancer Activities and Drug Delivery Systems of Tannins. *Medicinal Research Reviews*.

[81] Lee S. K., Kim J. H., Kim J. S. (2012). Polyphenol (−)-epigallocatechin gallate-induced cardioprotection may attenuate ischemia-reperfusion injury through adenosine receptor activation: a preliminary study. *Korean Journal of Anesthesiology*.

[82] Gokulakrisnan A., Jayachandran Dare B., Thirunavukkarasu C. (2011). Attenuation of the cardiac inflammatory changes and lipid anomalies by (−)-epigallocatechin-gallate in cigarette smoke-exposed rats. *Molecular and Cellular Biochemistry*.

[83] Koo S. I., Noh S. K. (2007). Green tea as inhibitor of the intestinal absorption of lipids: potential mechanism for its lipid-lowering effect. *Journal of Nutritional Biochemistry*.

[84] Jang Y. H., Lee Y. C., Park N. H. (2006). Polyphenol (−)-epigallocatechin gallate protection from ischemia/reperfusion-induced renal injury in normotensive and hypertensive rats. *Transplantation Proceedings*.

[85] Wang S., Noh S. K., Koo S. I. (2006). Green tea catechins inhibit pancreatic phospholipase A_2_ and intestinal absorption of lipids in ovariectomized rats. *The Journal of Nutritional Biochemistry*.

[86] Jin Y.-R., Im J.-H., Park E.-S. (2008). Antiplatelet activity of epigallocatechin gallate is mediated by the inhibition of PLC*γ*2 phosphorylation, elevation of PGD2 production, and maintaining calcium-ATPase activity. *Journal of Cardiovascular Pharmacology*.

[87] Kang W.-S., Lim I.-H., Yuk D.-Y. (1999). Antithrombotic activities of green tea catechins and (−)-epigallocatechin gallate. *Thrombosis Research*.

[88] Skovronsky D. M., Lee V. M.-Y., Trojanowski J. Q. (2006). Neurodegenerative diseases: new concepts of pathogenesis and their therapeutic implications. *Annual Review of Pathology*.

[89] Lee J. W., Lee Y. K., Ban J. O. (2009). Green tea (−)-epigallocatechin-3-gallate inhibits *β*-amyloid-induced cognitive dysfunction through modification of secretase activity via inhibition of ERK and NF-*κ*B pathways in mice. *Journal of Nutrition*.

[90] Giunta B., Hou H., Zhu Y. (2010). Fish oil enhances anti-amyloidogenic properties of green tea EGCG in Tg2576 mice. *Neuroscience Letters*.

[91] Weinreb O., Mandel S., Amit T., Youdim M. B. H. (2004). Neurological mechanisms of green tea polyphenols in Alzheimer's and Parkinson's diseases. *Journal of Nutritional Biochemistry*.

[92] Ortiz-López L., Márquez-Valadez B., Gómez-Sánchez A. (2016). Green tea compound epigallo-catechin-3-gallate (EGCG) increases neuronal survival in adult hippocampal neurogenesis in vivo and in vitro. *Neuroscience*.

[93] Zhang B., Wang B., Cao S., Wang Y. (2015). Epigallocatechin-3-gallate (EGCG) attenuates traumatic brain injury by inhibition of edema formation and oxidative stress. *The Korean Journal of Physiology & Pharmacology*.

[94] Scholey A., Downey L. A., Ciorciari J. (2012). Acute neurocognitive effects of epigallocatechin gallate (EGCG). *Appetite*.

[95] Nance C. L., Siwak E. B., Shearer W. T. (2009). Preclinical development of the green tea catechin, epigallocatechin gallate, as an HIV-1 therapy. *Journal of Allergy and Clinical Immunology*.

[96] Li S., Hattori T., Kodama E. N. (2011). Epigallocatechin gallate inhibits the HIV reverse transcription step. *Antiviral Chemistry and Chemotherapy*.

[97] Wang Y., Li J., Wang X. (2016). (−)-Epigallocatechin-3-Gallate Enhances Hepatitis C Virus Double-Stranded RNA Intermediates-Triggered Innate Immune Responses in Hepatocytes. *Scientific Reports*.

[98] Xu J., Gu W., Li C. (2016). Epigallocatechin gallate inhibits hepatitis B virus via farnesoid X receptor alpha. *Journal of Natural Medicines*.

[99] Zhao M., Jiang J., Zheng R. (2012). A proprietary topical preparation containing EGCG-stearate and glycerin with inhibitory effects on herpes simplex virus: Case study. *Inflammation and Allergy - Drug Targets*.

[100] Kim M., Kim S.-Y., Lee H. W. (2013). Inhibition of influenza virus internalization by (−)-epigallocatechin-3- gallate. *Antiviral Research*.

[101] Weber J. M., Ruzindana-Umunyana A., Imbeault L., Sircar S. (2003). Inhibition of adenovirus infection and adenain by green tea catechins. *Antiviral Research*.

[102] Song J.-M., Lee K.-H., Seong B.-L. (2005). Antiviral effect of catechins in green tea on influenza virus. *Antiviral Research*.

[103] Ho H.-Y., Cheng M.-L., Weng S.-F., Leu Y.-L., Chiu D. T.-Y. (2009). Antiviral effect of epigallocatechin gallate on enterovirus 71. *Journal of Agricultural and Food Chemistry*.

[104] Calland N., Dubuisson J., Rouillé Y., Séron K. (2012). Hepatitis C virus and natural compounds: A new antiviral approach?. *Viruses*.

[105] Friedman M. (2007). Overview of antibacterial, antitoxin, antiviral, and antifungal activities of tea flavonoids and teas. *Molecular Nutrition & Food Research*.

[106] Steinmann J., Buer J., Pietschmann T., Steinmann E. (2013). Anti-infective properties of epigallocatechin-3-gallate (EGCG), a component of green tea. *British Journal of Pharmacology*.

[107] Gordon N. C., Wareham D. W. (2010). Antimicrobial activity of the green tea polyphenol (−)-epigallocatechin-3-gallate (EGCG) against clinical isolates of *Stenotrophomonas maltophilia*. *International Journal of Antimicrobial Agents*.

[108] Kono K., Tatara I., Takeda S., Arakawa K., Hara Y. (1994). Antibacterial activity of epigallocatechin gallate against methicillin-resistant *Staphylococcus aureus*. *Kansenshogaku Zasshi. The Journal of the Japanese Association for Infectious Diseases*.

[109] Farhad Mollashahi N., Bokaeian M., Farhad Mollashahi L., Afrougheh A. (2015). Antifungal efficacy of green tea extract against candida albicans biofilm on tooth substrate. *Journal of Dentistry (Tehran, Iran)*.

[110] Hirasawa M., Takada K. (2004). Multiple effects of green tea catechin on the antifungal activity of antimycotics against Candida albicans. *Journal of Antimicrobial Chemotherapy*.

[111] Baizabal-Aguirre V. M., Rosales C., López-Macías C., Gómez M. I. (2014). Control and resolution mechanisms of the inflammatory response. *Mediators of Inflammation*.

[112] Scott D. L., Wolfe F., Huizinga T. W. J. (2010). Rheumatoid arthritis. *The Lancet*.

[113] Firestein G. S. (2003). Evolving concepts of rheumatoid arthritis. *Nature*.

[114] Ahmed S., Rahman A., Hasnain A., Lalonde M., Goldberg V. M., Haqqi T. M. (2002). Green tea polyphenol epigallocatechin-3-gallate inhibits the IL-1*β*-induced activity and expression of cyclooxygenase-2 and nitric oxide synthase-2 in human chondrocytes. *Free Radical Biology and Medicine*.

[115] Morinobu A., Biao W., Tanaka S. (2008). (−)-Epigallocatechin-3-gallate suppresses osteoclast differentiation and ameliorates experimental arthritis in mice. *Arthritis and Rheumatism*.

[116] Lee S.-Y., Jung Y. O., Ryu J.-G. (2016). Epigallocatechin-3-gallate ameliorates autoimmune arthritis by reciprocal regulation of T helper-17 regulatory T cells and inhibition of osteoclastogenesis by inhibiting STAT3 signaling. *Journal of Leukocyte Biology*.

[117] Riegsecker S., Wiczynski D., Kaplan M. J., Ahmed S. (2013). Potential benefits of green tea polyphenol EGCG in the prevention and treatment of vascular inflammation in rheumatoid arthritis. *Life Sciences*.

[118] Singh A. K., Umar S., Riegsecker S., Chourasia M., Ahmed S. (2016). Regulation of Transforming Growth Factor *β*-Activated Kinase Activation by Epigallocatechin-3-Gallate in Rheumatoid Arthritis Synovial Fibroblasts: Suppression of K63-Linked Autoubiquitination of Tumor Necrosis Factor Receptor-Associated Factor 6. *Arthritis and Rheumatology*.

[119] Singh R., Ahmed S., Islam N., Goldberg V. M., Haqqi T. M. (2002). Epigallocatechin-3-gallate inhibits interleukin-1*β*-induced expression of nitric oxide synthase and production of nitric oxide in human chondrocytes: Suppression of nuclear factor *κ*B activation by degradation of the inhibitor of nuclear factor *κ*B. *Arthritis and Rheumatism*.

[120] Moon H.-S., Lee H.-G., Choi Y.-J., Kim T.-G., Cho C.-S. (2007). Proposed mechanisms of (−)-epigallocatechin-3-gallate for anti-obesity. *Chemico-Biological Interactions*.

[121] Thielecke F., Boschmann M. (2009). The potential role of green tea catechins in the prevention of the metabolic syndrome—a review. *Phytochemistry*.

[122] Raederstorff D. G., Schlachter M. F., Elste V., Weber P. (2003). Effect of EGCG on lipid absorption and plasma lipid levels in rats. *Journal of Nutritional Biochemistry*.

[123] Forester S. C., Gu Y., Lambert J. D. (2012). Inhibition of starch digestion by the green tea polyphenol, (−)-epigallocatechin-3-gallate. *Molecular Nutrition and Food Research*.

[124] Park J.-H., Bae J.-H., Im S.-S., Song D.-K. (2014). Green tea and type 2 diabetes. *Integrative Medicine Research*.

[125] Zuo X., Tian C., Zhao N. (2014). Tea polyphenols alleviate high fat and high glucose-induced endothelial hyperpermeability by attenuating ROS production via NADPH oxidase pathway. *BMC Research Notes*.

[126] Wolfram S., Raederstorff D., Preller M. (2006). Epigallocatechin gallate supplementation alleviates diabetes in rodents. *Journal of Nutrition*.

[127] Tsuneki H., Ishizuka M., Terasawa M., Wu J.-B., Sasaoka T., Kimura I. (2004). Effect of green tea on blood glucose levels and serum proteomic patterns in diabetic (db/db) mice and on glucose metabolism in healthy humans. *BMC Pharmacology*.

[128] Wu L.-Y., Juan C.-C., Hwang L. S., Hsu Y.-P., Ho P.-H., Ho L.-T. (2004). Green tea supplementation ameliorates insulin resistance and increases glucose transporter IV content in a fructose-fed rat model. *European Journal of Nutrition*.

[129] Ortsäter H., Grankvist N., Wolfram S., Kuehn N., Sjöholm Å. (2012). Diet supplementation with green tea extract epigallocatechin gallate prevents progression to glucose intolerance in db/db mice. *Nutrition and Metabolism*.

[130] Waltner-Law M. E., Wang X. L., Law B. K., Hall R. K., Nawano M., Granner D. K. (2002). Epigallocatechin gallate, a constituent of green tea, represses hepatic glucose production. *Journal of Biological Chemistry*.

[131] Cai E. P., Lin J.-K. (2009). Epigallocatechin gallate (EGCG) and rutin suppress the glucotoxicity through activating IRS2 and AMPK signaling in rat pancreatic *β* cells. *Journal of Agricultural and Food Chemistry*.

[132] Withers D. J., Gutierrez J. S., Towery H., Bonner-Weir S., White M. F. (1998). Disruption of IRS-2 causes type 2 diabetes in mice. *Nature*.

[133] Deng Y.-T., Chang T.-W., Lee M.-S., Lin J.-K. (2012). Suppression of free fatty acid-induced insulin resistance by phytopolyphenols in C2C12 mouse skeletal muscle cells. *Journal of Agricultural and Food Chemistry*.

[134] Dong Z. (2000). Effects of food factors on signal transduction pathways. *BioFactors*.

[135] Sanvicens N., Marco M. P. (2008). Multifunctional nanoparticles—properties and prospects for their use in human medicine. *Trends in Biotechnology*.

[136] Huo C., Wan S. B., Lam W. H. (2008). The challenge of developing green tea polyphenols as therapeutic agents. *Inflammopharmacology*.

[137] Müller R. H., Mäder K., Gohla S. (2000). Solid lipid nanoparticles (SLN) for controlled drug delivery—a review of the state of the art. *European Journal of Pharmaceutics and Biopharmaceutics*.

[138] Weiss J., Takhistov P., McClements D. J. (2006). Functional materials in food nanotechnology. *Journal of Food Science*.

[139] Üner M. (2006). Preparation, characterization and physico-chemical properties of solid lipid nanoparticles (SLN) and nanostructured lipid carriers (NLC): their benefits as colloidal drug carrier systems. *Pharmazie*.

[140] Gerasimov O. V., Boomer J. A., Qualls M. M., Thompson D. H. (1999). Cytosolic drug delivery using pH- and light-sensitive liposomes. *Advanced Drug Delivery Reviews*.

[141] Voinea M., Simionescu M. (2002). Designing of ‘intelligent’ liposomes for efficient delivery of drugs. *Journal of Cellular and Molecular Medicine*.

[142] Soppimath K. S., Aminabhavi T. M., Kulkarni A. R., Rudzinski W. E. (2001). Biodegradable polymeric nanoparticles as drug delivery devices. *Journal of Controlled Release*.

[143] Delie F., Blanco-Príeto M. J. (2005). Polymeric particulates to improve oral bioavailability of peptide drugs. *Molecules*.

[144] Gref R., Gref R., Minamitake Y. (1994). Biodegradable long-circulating polymeric nanospheres. *Science*.

[145] Wang S., Su R., Nie S. (2014). Application of nanotechnology in improving bioavailability and bioactivity of diet-derived phytochemicals. *Journal of Nutritional Biochemistry*.

