# Supplementary figures and images for: Therapeutic Potential of Epigallocatechin Gallate Nanodelivery Systems
Source: Biomed Res Int. 2017 Jul 16;2017:5813793. doi: 10.1155/2017/5813793 (PMC5534279; doi:10.1155/2017/5813793)

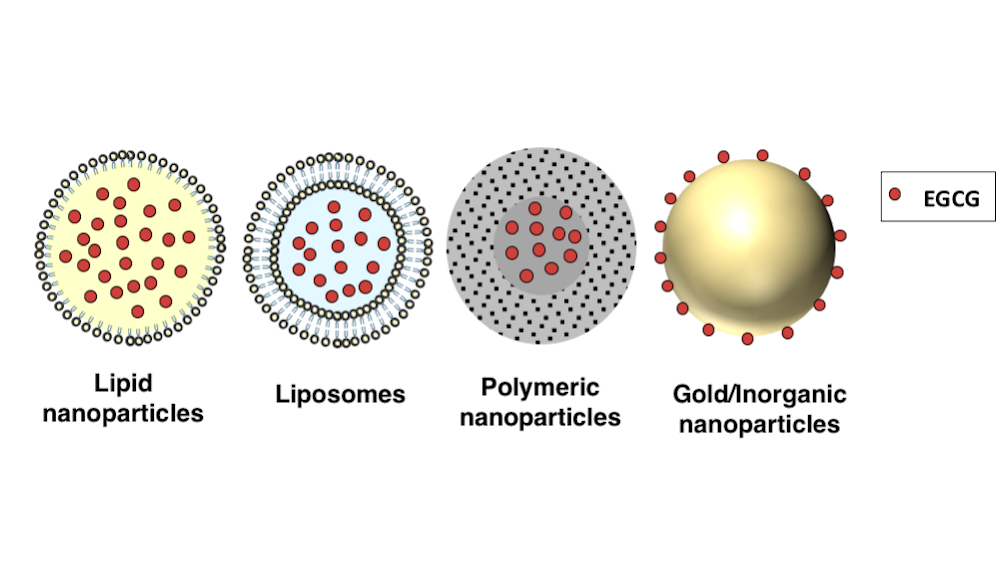

Supplement: Supplementary file 1 — Figure S1. Different types of nanoparticles used as delivery vehicles of EGCG. [file 5813793.f1.tiff]
